# Measuring Scope of Practice Enactment Among Primary Care Registered Nurses

**DOI:** 10.1177/08445621211058328

**Published:** 2021-11-20

**Authors:** Suzanne Braithwaite, Joan Tranmer, Rosemary Wilson, Joan Almost, Deborah Tregunno

**Affiliations:** 1 School of Nursing, 4257 Queen's University; 2 School of Health Human and Justice Studies, 49998 Loyalist College

**Keywords:** Primary care, Family practice, Nurses, Scope of practice, Nurse roles, Survey designs

## Abstract

**Background:**

Scope of practice enactment is poorly understood in the primary care setting.

**Purpose:**

The following research objectives were addressed: (1) to revise and adapt the Actual Scope of Practice (ASCOP) questionnaire for use in the primary care setting, and (2) to determine internal consistency, construct validity, and sensitivity of the modified instrument.

**Methods:**

To address the first objective, a narrative literature review and synthesis and an expert panel review was conducted. To address the second objective, a cross-sectional survey of 178 registered nurses who worked in primary care was conducted.

**Results:**

The ASCOP, with few modifications, addressed key attributes of nursing scope of practice in the primary care setting. The modified instrument yielded acceptable alpha coefficients ranging from 0.66 to 0.91. Total mean score of 4.8 (SD  =  .67) suggests that registered nurses within interprofessional primary care teams almost always engage in activities reflected in the modified instrument.

**Conclusions:**

The modified instrument is the first instrument validated to measure nursing scope of practice enactment in the primary care setting. Findings from this study support the use of the modified ASCOP questionnaire as a reliable and valid measure of scope of practice enactment among primary care registered nurses.

## Background and purpose

Primary care is the point of entry into the health care system. It is the setting in which new health needs are identified and addressed, and continuing care is provided over time (Starfield, 1994). Primary care providers are responsible for coordinating and integrating care between organizations and specialists (Starfield, 1994). Services within primary care are a component of primary health care, a broader concept that refers to an approach to health and a spectrum of services beyond traditional health care systems (Government of Canada, 2012).

In Canada, many provinces have sought to expand primary care services by implementing new interprofessional primary care team delivery models. In Ontario, the focus has been on structural changes such as new payment structures and practice models. In the 1980s, community health centres (CHC) emerged in Ontario as an interprofessional model of primary care to meet the unique needs of high-risk populations (Collins et al., 2014). Community health centres are characterized by community governance, a focus on social determinants of health and health promotion, community outreach, and salaried interprofessional teams (Collins et al., 2014). Along with CHCs, new models have emerged including family health teams (FHT) and nurse practitioner-led clinics (NPLC). These collaborative interprofessional models of care were created with the intention to expand access to person-centered and coordinated comprehensive primary care services to ensure Ontarians receive the care they need in their communities (Ministry of Health & Long Term Care, 2010, 2011, 2015). Teams include nurses, physicians, pharmacists, dietitians, social workers, and other health care providers.

Registered nurses (RN) are well qualified to provide high quality, primary care services within the framework of primary health care (Besner, 2006; Griffiths & Murrells, 2011; Lukewich et al., 2016; Turley et al., 2018). However, the roles of nurses in primary care settings are not well understood (Norful et al., 2017; Oelke et al., 2014; Turley et al., 2018). And currently, we have no capacity to measure scope of practice enactment in the primary care setting. Thus, we are unable to adequately assess or articulate the extent to which RNs apply the breadth of their professional scope of practice, and appropriately address potential underutilization of the nursing workforce.

### Scope of practice enactment

The concept ‘scope of practice enactment’ relies on the broader concept, full scope of practice. The International Council of Nurses (ICN) defines nursing as “the promotion of health, prevention of illness, and the care of ill, disabled and dying people. Advocacy, promotion of safe environment, research, participation in shaping health policy and in patient and health systems management and education are also key nursing roles” (International Council of Nurses, 2002). In Canada, full scope of practice can be conceptually defined as the outer limits of practice all RNs are educated and authorized to perform that is influenced by the setting in which the nurse practices, the needs of the patients, and the knowledge and skill of the individual nurse (Almost, 2021). Full scope of practice is a role that is reflected in the knowledge base of the profession (Almost, 2021; Besner, 2006). Role enactment is a concept that is most closely related to scope of practice enactment and refers to the application of behaviour and knowledge as defined by cultural and professional expectations (Besner, 2005). Role enactment refers specifically to the application of the expected role, as defined by the individual organization, culture, and nurse (Déry et al., 2015; D’Amour et al., 2012). Drawing from the work of D’Amour et al. (2012), Déry et al. (2015), Canadian Nurses Association (Almost, 2021), and the ICN (International Council of Nurses, 2002), scope of practice enactment is conceptually defined as the extent to which RNs apply in practice the breadth of the professional scope of practice all RNs are educated and authorized to perform. Enactment speaks specifically to application of the knowledge base of the profession.

### Actual scope of practice questionnaire

The Actual Scope of Practice (ASCOP) questionnaire is a tool used to measure scope of practice enactment in the hospital setting (D’Amour et al., 2012). The questionnaire was developed by Canadian researchers and, to our knowledge, is the only published instrument that measures scope of practice enactment in nursing. The ASCOP questionnaire is a self-administered questionnaire consisting of 26 questions measuring scope of practice enactment rated on a six-point Likert-scale ranging from never to always. D’Amour et al. (2012) conducted a content analysis of the literature addressing scope of practice and defined six dimensions of nurses’ scope which reflect key nursing competencies (Besner, 2005; Black et al., 2008; Canadian Family Practice Nurses Association, 2019; Canadian Nurses Association, 2015). The competencies include, (1) assessment and care planning, (2) teaching of patients and families, (3) communication and care coordination, (4) integration and supervision of staff, (5) quality of care and patient safety, and (6) knowledge updating and utilization. Each item in the questionnaire is assigned a level of complexity from one to three. Level one items represent activities of low complexity, level two items represent moderate complexity, and level three items represent activities involving a high degree of complexity.

D’Amour et al. (2012) established the ASCOP questionnaire as a valid and reliable instrument for measuring scope of practice enactment in the acute care setting. However, scope of practice enactment has yet to be measured in the primary care setting. Measuring scope of practice enactment would provide nursing researchers, managers, and leaders with a means to measure, assess, and articulate the extent to which RNs apply the breadth of their professional scope of practice; resulting in the potential to influence research, practice development initiatives, organizational support, and policy change.

### Study aim

The overall aim of this study was to measure scope of practice enactment in the primary care setting. Two research objectives were addressed: (1) to adapt and revise the ASCOP questionnaire to measure scope of practice enactment in the primary care setting in Canada, and (2) to determine internal consistency, construct validity, and sensitivity of the modified instrument.

## Methods and procedures

To address the stated research objectives, this study consisted of two phases: (1) literature review and synthesis and expert panel review; and (2) cross-sectional survey of RNs working in interprofessional primary care (IPC) organizations.

### Phase One: literature review and synthesis and expert panel review

A descriptive narrative literature review was conducted to establish content validity of the ASCOP questionnaire for use in Canada's primary care setting. The purpose of the review and synthesis was to assess the extent to which items from the ASCOP questionnaire reflect the RN role in the context of primary care nursing. The objectives of the review therefore were to (1) synthesize the evidence regarding the role of RNs in Canada's primary care setting, (2) map role activities to the dimensions within the ASCOP questionnaire, and (3) modify the existing ASCOP to reflect nursing and the primary care context.

We searched English literature from 2005 to 2015 in CINAHL, PsychINFO, MEDLINE and EMBASE databases using the following keywords: nurse, RN, primary care, family practice, physician's office, clinic, role, and scope of practice. The search was further limited to articles published in Canada due to the socio-political nature of nursing scope of practice across national borders. A total of 181 citations were retrieved. Papers were also obtained through cross-checking of reference lists of identified studies, as well as hand searching unpublished studies, grey literature, and government and societal websites. Due to the limited body of literature on this topic, qualitative and quantitative studies, grey literature, and government and organization websites were included in this review. A total of 12 papers/reports were included in the review. Of these, 4 were reports from professional nursing associations (Canadian Family Practice Nurses; Association, 2012; College & Association of Registered Nurses of Alberta, 2011; Martin-Misener & Bryant-Lukosius, 2014; Registered Nurses’ Association of Ontario, 2012). Subsequently, the content of the ASCOP questionnaire was reviewed and modified as necessary, to reflect the nursing role in the primary care setting. The modified ASCOP is referred to as the Actual Scope of Practice-Primary Care (ASCOP-PC) questionnaire.

We presented the ASCOP-PC questionnaire to a panel of four experts in primary care, and one expert in survey-based research methods. Using an electronic web-based platform (Fluids Survey©), the panel was asked to rate each item on a scale of one to four based on content, clarity, and comprehension. Reviewers were also asked to add written feedback. Questions receiving a mean rate lower than three were revised based on the recommendations, and the process was repeated until an item-level content validity index (I-CVI) of greater than 0.80 was achieved (Polit & Beck, 2017).

### Phase Two: cross-sectional survey

We conducted a cross-sectional survey to assess construct validity, internal consistency, and sensitivity of the ASCOP-PC questionnaire in the primary care setting. Five local primary care RNs were asked to pilot the survey package. No items on the questionnaire were modified as a result of the pilot test and responses from the pilot study participants were not included in the final analysis of the cross-sectional survey. The ASCOP-PC items are presented in the Supplemental Material 1.

#### Sample

A list of interprofessional primary care (IPC) teams was retrieved from the Ontario Ministry of Health and Long-Term Care. A total of 284 IPC teams were operating in Ontario (a large province in Canada) in 2015, including 77 Community Health Centers (CHC), 182 Family Health Teams (FHT), and 25 Nurse Practitioner-Led Clinics (NPLC). Attempts were made to contact all 284 organizations and contact was made with 158 organizations (55.6%). A total of 210 RNs were employed by the IPC organizations. A nursing or clinical lead from each organization was contacted by the researcher via telephone, introduced to the study, and invited to distribute the electronic survey to the RN staff. The lead was asked to track and submit the number of RNs who received the survey to provide the researchers a means to capture response rate. The questionnaire was delivered to 210 RNs.

#### Data collection

The ASCOP-PC questionnaire along with participant demographics were formatted into a web-based electronic platform (Fluids Survey ©). Dillman's (2000) recommendations for web-based survey research methods were used during development of the self-administered web-based survey package. The survey was distributed in the fall of 2015. Two weeks following the initial distribution of the questionnaire, an email was sent to remind participants to complete the questionnaire. A second reminder email was sent out after an additional two weeks. A letter of information and consent was included on the first page of the survey and participants indicated their consent prior to advancing in the survey.

#### Statistical analysis

Our data analysis plan was informed by a similar study conducted by D’Amour et al. (2012), and data were analyzed using SPSS© (version 23) statistical analysis software. To assess internal consistency and validity of the instrument we employed a descriptive and factor analysis approach. Because the ASCOP-PC questionnaire items are grouped into six dimensions (subscales), principal component analysis (PCA) is an appropriate data analysis approach. We conducted PCA for each subscale and overall scale to measure the proportion of variance explained by the grouping of items.

To determine instrument sensitivity and construct validity, we carried out t-tests, comparing total and subscale scores for variables with two categorical levels, to test the instrument's ability to distinguish between known-groups. Additionally, analysis of variance and Tukey post hoc tests were computed for variables with greater than 2 levels. Statistical significance was set at P < 0.05. The groups analyzed include (1) years of nursing experience, (2) years of experience in primary care, (3) nursing education, (4) practice role, (5) employment status, and (6) organizational structure.

## Results

### Phase One: literature review and expert panel review

Content extracted from the literature was summarized and compared to items from the questionnaire. The literature was categorized according to the six dimensions of practice from the ASCOP questionnaire. All six dimensions of practice were reflected in three of the articles included in the review. The dimensions *assessment and care planning* and teaching of patients and families were relevant themes throughout the literature and were represented in 11 of the 12 articles reviewed. *Communication and care coordination* was represented in eight articles, *knowledge updating and utilization* and *integration and supervision of staff* were each represented in five articles. *Quality of care and patient safety* was represented in four articles. All items in the questionnaire were represented in the literature. No items were removed from the questionnaire and one new item was added under the *assessment and care planning* domain. The new item reflected a dimension of nursing practice that was present in 10 of the articles reviewed.

The instrument underwent three rounds of review with the panel of experts. After the first review, 20 items were modified, no items were removed. An additional four items were subsequently modified after the second review. The third and final review yielded mean rates greater than three on all items. In newly developed tools it is recommended that an I-CVI value of at least 0.78 be achieved and a scale-level content validity index (S-CVI) greater than 0.90 be achieved (Polit & Beck, 2017). All I-CVIs calculated were greater than 0.78 and the S-CVI was 0.95 which were considered acceptable. No items were modified during the third review.

### Phase Two: cross-sectional survey

Of the 210 RNs who received the survey, 181 were completed, providing an 86.2% response rate. Three questionnaires completed by NPs were excluded from the analysis as not meeting the inclusion criteria, leaving 178 participants included in the analysis. Demographic characteristics of the sample are displayed in Table 1. Of the participants, 87.1% were female compared to 93.9% in the general RN population in Ontario in 2015 (College of Nurses of Ontario, 2016). 62.6% of the participants had a baccalaureate degree or higher, and only 16.5% had practiced in primary care for more than 10 years. Majority of respondents worked in a FHT (61.8%) or a CHC (21.4%).

**Table 1.** Demographic Characteristics of the Study Population.

	Mean	Median	Range
Age (years)	43.2	43	22-71
Years of Nursing Experience	18.0	15.5	1-50
Years of Experience in Primary Care	7.0	5.0	1-44
		**Responses, n (%)**	
**Sex**			
Male		6 (3.4)	
Female		155 (87.1)	
No response		17 (9.6)	
**Current role**			
Staff nurse		123 (73.2)	
Other		45 (26.8)	
**Employment status** ^a^			
Full time		125 (74.4)	
Other		43 (25.6)	
**Locality** ^b^			
Urban/City		96 (55.5)	
Rural		71 (41.0)	
Remote		6 (3.5)	
**Also working outside of primary care** ^c^			
Yes		35 (21.0)	
No		132 (79.0)	
**Organizational structure** ^b^			
Community Health Centre		37 (21.4)	
Family Health Team		107 (61.8)	
NPLC		9 (5.2)	
Other		20 (11.6)	
**Highest level of education** ^d^			
Diploma		61 (37.4)	
Baccalaureate or higher		102 (62.6)	

^a^Missing n = 10.

^b^Missing n = 5.

^c^Missing n = 11.

^d^Missing n = 15.

As shown in Table 2, the overall item mean score was 4.8 out of 6.0, and item mean scores ranged from 2.9 to 6.0 (µ3.1). Subscale mean scores ranged from 4.2 to 5.2, and levels of complexity mean scores ranged from 4.5 to 5.3. The highest dimension scores were teaching of patients and families (5.2 + /-0.7), and knowledge utilization and updating (5.1 + /-0.7). Those dimensions less often reported include assessment and care planning (4.8 + /-0.7), communication and care coordination (4.9 + /-0.8), and quality of care and patient safety (4.7 + /-1.0). The dimension least frequently reported was integration and supervision of staff (4.2 + /-1.1).

**Table 2.** Profile of Scores: Dimensions and Levels of Complexity.

	Dimensions								Levels of Complexity		
	Global Mean Score	Assessment and care planning	Teaching of patients and families	Communication and care coordination	Integration and supervision of staff	Quality of care and patient safety	Knowledge utilization and updating		1	2	3
Number of Items	26	6	4	5	4	5	3		7	10	10
Mean	4.81	4.83	5.16	4.86	4.20	4.66	5.12		5.31	4.76	4.49
SD	0.668	0.749	0.725	0.824	1.174	1.00	0.747		0.597	0.697	0.917
Median	4.84	4.83	5.25	5.00	4.33	4.75	5.33		5.43	4.90	4.50
Range	3.10(2.9-6.0)	3.40(2.6-3.4)	3.00(3.0-6.0)	3.60(2.4-6.0)	5.00(1.0-6.0)	4.75(1.3-6.0)	3.33(2.7-6.0)		2.57(3.4-6.0)	3.40(2.6-6.0)	4.56(1.4-6.0)

#### Construct validity

Descriptive and comparative data analyses for known-groups are presented in Supplemental Material 2. Scores on the ASCOP-PC varied little across the known sub groups. Nurses with less than five years of experience in primary care scored lower in integration and supervision of staff (P  =  .011, η^2^  =  .016). The group categorized as staff nurses scored lower in the assessment and care planning dimension (P  =  .004, η^2^  =  .051) as well as lower scores for level of complexity (P  =  .009, η^2^  =  .02) when compared with nurses in the “other” category (including clinical coordinators, managers, and educators). No differences were identified in the following groups: years of experience in nursing, nursing education, employment status, or organizational structure.

#### Internal consistency and validity

The Kaiser-Meyer-Olkin (KMO) measure verified the sampling adequacy for the analysis, KMO  =  .83 and all KMO values for the 6 subscales were >.625 which is well above the acceptable limit of .5 (Field, 2018). Bartlett's test of sphericity X2 (351)  =  2090.344, p  =  < .001 indicated that correlations between items were sufficiently large for PCA. As presented in Supplemental Material 2, the PCA for each dimension explained variances ranging from 44.2% to 62.6%, and 59.8% for the instrument as a whole. The alpha coefficient for the 27 items together was .91, demonstrating acceptable internal consistency. Two of the dimensions had adequate internal consistency with α coefficients of .75 and .79, while values for the remaining four dimensions range from .66 to .68, demonstrating only modest internal consistency within the dimensions.

## Discussion

In this study we conducted initial psychometric testing of the ASCOP-PC questionnaire in the primary care setting. A content and context relevant questionnaire was developed from findings of a comparative content review and expert panel feedback. Based on our findings, we would suggest that the ASCOP-PC questionnaire is a consistent and reliable measure of nursing scope of practice enactment in the primary care setting.

The ASCOP-PC contained valid content as determined by our comparative content review and validation by a panel of experts. Our psychometric testing determined that the tool measured important primary care nursing constructs and had good internal consistency. Internal consistency of the modified questionnaire was high, with alpha coefficients of .91; however, four of the subscales only demonstrated moderate internal consistency with alpha coefficients < .70. Generally, an alpha coefficient of .70 or higher is considered acceptable (Tabachnick & Fidell, 2007); however, an alpha coefficient between .60 and .70 is considered acceptable when conducting initial psychometric testing of an instrument (Loewenthal, 2004). The alpha coefficients for individual dimensions (Cronbach α < .70) provide moderate support for internal validity with the proposed dimension structure. Four of the six dimensions demonstrated alpha coefficients below .70 including teaching of patients and families, knowledge updating and utilization, assessment and care planning, and communication and care coordination. Low alpha coefficients may be related to the low number of items within the dimensions (Tavakol & Dennick, 2011). Further, low alpha coefficients can occur when the dimension being analyzed has additional underlying factors or dimensions (Tavakol & Dennick, 2011). The ASCOP-PC categorizes items in two ways, (1) by the dimension of nursing practice it reflects, and (2) according to the level of complexity of the activity. It is possible that the level of complexity of each item may represent a separate dimension structure influencing the alpha coefficient. Subsequent research should seek to establish a dimension structure for the ASCOP-PC that reflects the levels of complexity of the activities to further validate the proposed dimension structure.

Few differences in scope of practice enactment between known-groups were revealed in this study. One potential contributing factor is the poor understanding of primary care nursing. Without a clear understanding of nursing within this setting, it is difficult to establish “known-groups.” Further research is required to better understand the unique experience of RNs in primary care and to reveal and establish groups of nurses within the population that are likely to vary in scope of practice enactment. Conversely, working in team-based models of care, within an organization in which the executive director is willing to forward the survey may contribute to the potential homogeneity of the sample and less variance between known-groups.

### Similarities and differences: comparing the ASCOP-PC with the original ASCOP

This study was largely informed by the study conducted by D’Amour et al. (2012) in which the initial development and psychometric testing of the ASCOP was completed. Findings from D’Amour et al. (2012) are compared to findings from this study. A literature review and synthesis and expert panel review yielded few changes to the original ASCOP questionnaire. Items were altered to reflect language consistent with the role in primary care, and one item was added to expand on activities within the assessment and care planning dimension. The changes did not significantly alter the original meaning behind the item and/or the dimension. Our findings suggest that the original questionnaire adequately reflects key nursing competencies consistent in all areas across various nursing roles and care settings.

Findings from the PCA in this study are consistent with findings from D’Amour's study (2012) as listed in Table 3. Both studies have a very comparable alpha Cronbach for the instrument as a whole demonstrating acceptable internal consistency. Within dimensions, the ASCOP yielded alpha coefficients ranging from 0.61 to 0.76 and the ASCOP-PC yielded alpha coefficients ranging from 0.64 to 0.79 demonstrating modest to acceptable internal consistency within dimensions. Explained variances were also similar with explained variances for both instruments close to 60 percent.

**Table 3.** Alpha Coefficients and Factor Analysis for the Dimensions of the ASCOP-PC.

Dimensions (subscale)	Number of Items	Cronbach's α	% Explained Variance	KMO
Total for the 6 dimensions	27	0.91	59.8	0.858
Assessment and care planning	6	0.67	58.2	0.678
Teaching of patients and families	4	0.64	52.7	0.688
Communication and care coordination	5	0.68	44.2	0.725
Integration and supervision of staff	4	0.79	61.3	0.637
Quality of care and patient safety	5	0.75	51.2	0.790
Knowledge utilization and updating	3	0.66	62.6	0.671

*Barlett's Test of Sphericity P* *<* *.001.*

Variances among mean scores were found between this study and findings from D’Amour and colleagues’ study (2012). A comparison of mean scores for each dimension and for the instrument as a whole is illustrated in Supplementary Material 3. D’Amour and colleagues’ study found a mean score of 3.47 for the ASCOP instrument as a whole suggesting that most activities were performed infrequently. In contrast, we found that primary care RNs reported engaging in most activities on the ASCOP-PC almost always, enacting their full scope of practice. This finding is inconsistent with previous studies which suggest RNs in primary care practice significantly below their full scope of practice (Besner, 2005; D’Amour et al., 2012; Kennedy, 2014; J. Lukewich et al., 2014; Moaveni et al., 2010; Nathenson et al., 2007; Upenieks et al., 2007; White et al., 2008). Variances between the sampling plans for the two studies may be a factor influencing these findings. It is possible characteristics of organizational attributes may be a factor influencing scope of practice enactment. Nurses practicing in team-based models of care may be more likely to enact their full scope of practice. Further research is required to understand the unique characteristics of interprofessional primary care teams and if the organizational characteristics of this sample contribute to scope of practice enactment. Further population-based research and descriptive observational studies are needed to validate this finding.

### Strengths and limitations

This research was strengthened by a strong research design, involving a comprehensive approach to instrument validation. This design provided the opportunity to establish construct validity, content validity, internal validity, and responsiveness of the instrument. Few limitations were identified in this study including self-report response bias, sample representativeness, and sample size.

Research that relies solely on self-report measures is prone to self-report response bias (Waltz et al., 2010). Participants have a tendency to respond in a socially desirable way, particularly when the questions are of sensitive nature, and participants are hesitant to report behaviour that may be perceived as less desirable (Donaldson & Grant-Vallone, 2002). It is reasonable to assume results from this study may over-estimate actual nursing scope of practice enactment. This potentially limits the interpretation of the findings.

An additional limitation of the study is the representativeness of the sample. Our study was limited by a convenience sample of RNs practicing in organizations which were accessible by phone with a leader agreeable to forward the survey to RNs. Of the 2525 RNs practicing in the target organizations, the survey was only forwarded to 210 RNs (7.0%). Of those, 86.2% completed the survey. It is reasonable to assume that this sampling bias may have produced a sample with unique characteristics that are not necessarily representative of the population. Further, these team-based models of care may have more supports in place to engage in more nursing specific activities. These differences have been considered in the interpretation of this data. This limitation threatens the external validity of the study.

Reliability of factor analysis is dependent on sample size (Field, 2018), and although the minimal sample size required for factor analysis was obtained, the sample size is at the lower end of acceptable. The known-groups approach to establishing construct validity relies on prior research to identify groups that are expected to have differences in their responses. The known groups in this study were informed by a previous study in the acute care setting, and the limitation was considered in the interpretation of the results. Finally, the instrument was studied in one province within Canada and the results are limited to the unique social, political, and professional landscape of Ontario's primary care setting. This study requires further validation in other jurisdictions within Canada and internationally.

### Nursing practice, policy and research implications

Appropriate RN roles are needed to address the complex health needs of the aging population. The need for primary care nursing services is expected to grow as we face new population needs and health demands. Access to a RN in primary care provides patients with a range of care that meets their health needs and may significantly decrease referrals to more expensive levels of the health system. Findings from this research present a promising tool for nurses and leaders to assess or articulate the extent to which RNs apply the breadth of their professional scope of practice. The tool can be used by individual nurses as a self-evaluation tool and a driver to enhance their practice. Further, there is potential to address any underutilization of the nursing workforce that is uncovered. Nurses and leaders alike can leverage this tool to support optimization of the nursing role and effective utilization of scare health human resources.

This instrument requires further validation in other jurisdictions outside Ontario, Canada, and among RNs practicing in a variety of settings. It should be tested in primary care settings and organizations that differ from the collaborative interprofessional models studied in this project. To date, no study has quantitatively assessed the extent to which primary care RNs apply the breadth of their professional full scope of practice; therefore, the current state of scope of practice enactment in this setting is not fully understood. Our study suggests that the professional scope of practice of RNs is being effectively utilized within Ontario's interprofessional primary care teams. This finding requires additional research to adequately describe the extent to which RNs enact their full scope of practice in this setting.

Future studies may also compare nursing scope of practice enactment and organizational characteristics. This research would provide insight into the structures and processes that support or inhibit full scope of nursing practice. Findings from this could further support system redesign and provide nursing leaders with the knowledge to implement organizational interventions to enhance and support the role of nursing in primary care.

Further, researcher should seek to explore the relationship between nursing scope of practice enactment and patient health outcomes. It is important to understand the extent to which nursing scope of practice enactment may impact the health and wellbeing of patients. This research would provide significant direction for policy makers and decision makers in the reform of primary care services and organizational changes to support full scope of practice.

## Conclusion

The ASCOP-PC is a promising tool with potential to serve as an instrument to measure the extent to which RNs are applying the breadth of their professional scope of practice. The instrument could be used as a tool to measure system change and to identify the effectiveness of change efforts. Nursing leaders, administrators, and decision makers could use this tool to guide transformational change within the health system and to advance the development of primary care models that promote full scope of practice enactment, appropriate utilization of scarce resources, and effective team-based collaboration.

## Supplemental Material

sj-docx-1-cjn-10.1177_08445621211058328 - Supplemental material for Measuring Scope of Practice Enactment Among Primary Care Registered Nurses Click here for additional data file. Supplemental material, sj-docx-1-cjn-10.1177_08445621211058328 for Measuring Scope of Practice Enactment Among Primary Care Registered Nurses by Suzanne Braithwaite, Joan Tranmer, Rosemary Wilson, Joan Almost and Deborah Tregunno in Canadian Journal of Nursing Research

sj-docx-2-cjn-10.1177_08445621211058328 - Supplemental material for Measuring Scope of Practice Enactment Among Primary Care Registered Nurses Click here for additional data file. Supplemental material, sj-docx-2-cjn-10.1177_08445621211058328 for Measuring Scope of Practice Enactment Among Primary Care Registered Nurses by Suzanne Braithwaite, Joan Tranmer, Rosemary Wilson, Joan Almost and Deborah Tregunno in Canadian Journal of Nursing Research

sj-docx-3-cjn-10.1177_08445621211058328 - Supplemental material for Measuring Scope of Practice Enactment Among Primary Care Registered Nurses Click here for additional data file. Supplemental material, sj-docx-3-cjn-10.1177_08445621211058328 for Measuring Scope of Practice Enactment Among Primary Care Registered Nurses by Suzanne Braithwaite, Joan Tranmer, Rosemary Wilson, Joan Almost and Deborah Tregunno in Canadian Journal of Nursing Research

sj-pdf-4-cjn-10.1177_08445621211058328 - Supplemental material for Measuring Scope of Practice Enactment Among Primary Care Registered Nurses Click here for additional data file. Supplemental material, sj-pdf-4-cjn-10.1177_08445621211058328 for Measuring Scope of Practice Enactment Among Primary Care Registered Nurses by Suzanne Braithwaite, Joan Tranmer, Rosemary Wilson, Joan Almost and Deborah Tregunno in Canadian Journal of Nursing Research
