# Supplementary material for: Measuring Scope of Practice Enactment Among Primary Care Registered Nurses
Source: Can J Nurs Res. 2021 Nov 20;54(4):508–17. doi: 10.1177/08445621211058328 (PMC9597129; doi:10.1177/08445621211058328)
Supplement: sj-docx-1-cjn-10.1177_08445621211058328 - Supplemental material for Measuring Scope of Practice Enactment Among Primary Care Registered Nurses [file sj-docx-1-cjn-10.1177_08445621211058328.docx]

# Supplemental Material 1

ASCOP-PC Questionnaire Items, Dimensions, and Levels of Complexity

| Dimensions | # | Items | Levels of Complexity |
| --- | --- | --- | --- |
| Assessment and Care Planning | 25. | I autonomously assess the patient's physical and mental condition including taking biopsychosocial aspects into consideration. | 1 |
|  | 21. | I document information about the patient's condition (nursing assessment) and the care provided (therapeutic nursing plan, nurses' notes, etc.). | 1 |
|  | 2. | I use best practice guidelines and evidence-based assessment tools (pain scales, wound assessment tools, etc.) to plan nursing interventions in collaboration with patients and their family. | 2 |
|  | 1. | I develop patient care plans in collaboration with the patient, the patient's family, and the interprofessional care team. | 2 |
|  | 11. | I am involved in designing, applying, and evaluating/improving patient care programs. | 3 |
|  | 27. | I interpret assessment findings, coordinate/implement further nursing actions, and coordinate actions required by other members of the patient's health care team. | 1 |
| Teaching of Patients and Families | 10. | I assess the education needs of each patient and his/her family. | 1 |
|  | 17. | I assess the patient and family's comprehension/understanding of the teaching provided and tailor further educational interventions accordingly. | 1 |
|  | 5. | I use teaching strategies that are adapted to each patient and family in accordance with the patient’s current needs, intellectual competence, developmental stage, literacy level, etc.  I use teaching strategies that are adapted to each patient and family in accordance with the patient’s current needs, intellectual competence, developmental stage, literacy level, etc. | 2 |
|  | 22. | I assess and enhance (if applicable) the quality of patient education provided at my organization. | 3 |
| Communication and Care Coordination | 16. | I communicate all information that could affect the coordination of care with members of the patient's health care team. | 1 |
|  | 12. | I coordinate the care provided by the health care team to meet the needs of the patient and family. | 2 |
|  | 24. | I convey all relevant information to health care professionals in external organizations in order to ensure continuity of care. | 2 |
|  | 8. | I actively participate in interprofessional team meetings or activities. | 3 |
|  | 15. | To ensure continuity of care, I coordinate interventions of the patient's health care team within my organization and across sectors. | 3 |
| Integration and Supervision of Staff | 20. | I am involved in identifying in-service education needs for my organization. | 2 |
|  | 9. | I am involved in the orientation and training of nursing students and/or newly hired staff. | 2 |
|  | 6. | I act as a mentor or educator for newly hired staff. | 3 |
|  | 14. | I am involved in developing and conducting training activities for the health care team, in accordance with my skills. | 3 |
| Quality of Care and Patient Safety | 7. | I report clinical situations in which I see deficiencies in quality and safety of care. | 1 |
|  | 26. | I update clinical practices to improve the quality and safety of care. | 2 |
|  | 19. | When I have identified opportunities for improvement in care practices, I recommend approaches or strategies to improve the quality and safety of care provided within my organization. | 2 |
|  | 23. | I am involved in evaluating the quality and safety of care. | 3 |
|  | 3. | I am involved in developing and improving nursing practice to reflect evidence-based practice. | 3 |
| Knowledge Updating and Utilization | 4. | I keep my knowledge up-to-date. | 1 |
|  | 18. | I improve my practice based on new knowledge derived from best practices and research in nursing science or in health. | 2 |
|  | 13. | I share with the nursing team knowledge emerging from research. | 3 |
