# Supplementary material for: Measuring Scope of Practice Enactment Among Primary Care Registered Nurses
Source: Can J Nurs Res. 2021 Nov 20;54(4):508–17. doi: 10.1177/08445621211058328 (PMC9597129; doi:10.1177/08445621211058328)
Supplement: sj-docx-2-cjn-10.1177_08445621211058328 - Supplemental material for Measuring Scope of Practice Enactment Among Primary Care Registered Nurses [file sj-docx-2-cjn-10.1177_08445621211058328.docx]

# Supplemental Material 2

Known-groups Analysis

|  | | | Dimensions | | | | | | |  | Levels of Complexity | | |
| --- | --- | --- | --- | --- | --- | --- | --- | --- | --- | --- | --- | --- | --- |
|  | N | % | Global Mean Score | Assessment and care planning | Teaching of patients and families | Communication and care coordination | Integration and supervision of staff | Quality of care and patient safety | Knowledge utilization and updating |  | 1 | 2 | 3 |
| Years of nursing experience |  |  |  |  |  |  |  |  |  |  |  |  |  |
| <5 | 29 | 17.9 | 4.83 | 4.99 | 5.14 | 4.76 | 4.16 | 4.82 | 5.14 |  | 5.32 | 4.78 | 4.53 |
| 5-10 | 26 | 16.0 | 4.89 | 5.01 | 5.02 | 5.13 | 4.12 | 4.64 | 5.20 |  | 5.33 | 4.90 | 4.53 |
| 11-25 | 60 | 37.0 | 4.84 | 4.84 | 5.25 | 5.01 | 4.26 | 4.60 | 5.12 |  | 5.40 | 4.80 | 4.51 |
| >25 | 47 | 29.0 | 4.84 | 4.82 | 5.22 | 4.78 | 4.30 | 4.76 | 5.16 |  | 5.36 | 4.74 | 4.55 |
| Years of experience in primary care |  |  |  |  |  |  |  |  |  |  |  |  |  |
| <5 | 72 | 44.2 | 4.77 | 4.92 | 5.14 | 4.81 | 3.95 | 4.58 | 5.14 |  | 5.38 | 4.68 | 4.40 |
| 5-10 | 64 | 39.3 | 4.91 | 4.78 | 5.21 | 4.96 | **4.57^a^** | 4.83 | 5.16 |  | 5.31 | 4.89 | 4.64 |
| 11-25 | 24 | 14.7 | 4.98 | 5.12 | 5.26 | 5.19 | 4.19 | 4.80 | 5.19 |  | 5.51 | 4.93 | 4.66 |
| >25 | 3 | 1.8 | 4.92 | 4.60 | 5.75 | 4.80 | **4.78^a^** | 4.33 | 5.28 |  | 5.20 | 4.61 | 4.89 ^a^ |
| Nursing Education |  |  |  |  |  |  |  |  |  |  |  |  |  |
| Diploma | 61 | 37.4 | 4.86 | 4.78 | 5.29 | 4.97 | 4.30 | 4.74 | 5.16 |  | 5.39 | 4.74 | 4.58 |
| Baccalaureate  or higher | 100 | 62.6 | 4.85 | 4.96 | 5.13 | 4.90 | 4.21 | 4.69 | 5.15 |  | 5.35 | 4.80 | 4.54 |
| Current role |  |  |  |  |  |  |  |  |  |  |  |  |  |
| Staff nurse | 123 | 73.2 | 4.79 | 4.77 | 5.17 | 4.86 | 4.19 | 4.66 | 5.11 |  | 5.33 | 4.70 | 4.47 |
| Other | 45 | 26.4 | 5.01 | **5.15*** | 5.17 | 5.08 | 4.42 | 4.80 | 5.23 |  | 5.38 | **5.02*** | 4.72 |
| Employment Status |  |  |  |  |  |  |  |  |  |  |  |  |  |
| Full time | 125 | 74.4 | 4.81 | 4.85 | 5.17 | 4.88 | 4.15 | 4.67 | 5.12 |  | 5.33 | 4.77 | 4.48 |
| Other | 43 | 25.6 | 4.86 | 4.80 | 5.17 | 4.93 | 4.47 | 4.69 | 5.18 |  | 5.28 | 4.83 | 4.62 |
| Organizational Structure |  |  |  |  |  |  |  |  |  |  |  |  |  |
| CHC | 37 | 21.4 | 4.85 | 4.92 | 5.11 | 4.85 | 4.35 | 4.79 | 5.09 |  | 5.33 | 4.84 | 4.51 |
| FHT | 102 | 61.8 | 4.82 | 4.80 | 5.18 | 4.87 | 4.24 | 4.64 | 5.17 |  | 5.34 | 4.76 | 4.49 |
| NPLC | 9 | 5.2 | 4.58 | 4.56 | 5.21 | 4.89 | 3.35 | 4.45 | 4.94 |  | 5.10 | 4.51 | 4.25 |
| Other | 20 | 11.6 | 4.79 | 4.89 | 5.03 | 4.85 | 4.14 | 4.66 | 5.13 |  | 5.31 | 4.76 | 4.49 |

*Community Health Center (CHC); Family Health Team (FHT); Nurse Practitioner-Led Clinic (NPLC)*

*P<.05*
