# Supplementary material for: Measuring Scope of Practice Enactment Among Primary Care Registered Nurses
Source: Can J Nurs Res. 2021 Nov 20;54(4):508–17. doi: 10.1177/08445621211058328 (PMC9597129; doi:10.1177/08445621211058328)
Supplement: sj-docx-3-cjn-10.1177_08445621211058328 - Supplemental material for Measuring Scope of Practice Enactment Among Primary Care Registered Nurses [file sj-docx-3-cjn-10.1177_08445621211058328.docx]

# Supplemental Material 3

Comparison of ASCOP and ASCOP-PC

| Dimensions | Means | | Cronbach's α | | % Explained Variance | |
| --- | --- | --- | --- | --- | --- | --- |
|  | D’Amour | Braithwaite | D’Amour | Braithwaite | D’Amour | Braithwaite |
| Total for the 6 dimensions | 3.47 | 4.81 | 0.89 | 0.91 | 59.1 | 59.8 |
| Assessment and care planning | 4.19 | 4.83 | 0.64 | 0.67 | 43.0 | 58.2 |
| Teaching of patients and families | 3.88 | 5.16 | 0.67 | 0.64 | 54.5 | 52.7 |
| Communication and care coordination | 3.43 | 4.86 | 0.61 | 0.68 | 40.5 | 44.2 |
| Integration and supervision of staff | 3.03 | 4.20 | 0.70 | 0.79 | 52.9 | 61.3 |
| Quality of care and patient safety | 3.11 | 4.66 | 0.8 | 0.75 | 42.9 | 51.2 |
| Knowledge utilization and updating | 3.09 | 5.12 | 0.70 | 0.66 | 62.4 | 62.6 |
