# Supplementary material for: Measuring Scope of Practice Enactment Among Primary Care Registered Nurses
Source: Can J Nurs Res. 2021 Nov 20;54(4):508–17. doi: 10.1177/08445621211058328 (PMC9597129; doi:10.1177/08445621211058328)
Supplement: sj-pdf-4-cjn-10.1177_08445621211058328 - Supplemental material for Measuring Scope of Practice Enactment Among Primary Care Registered Nurses [file sj-pdf-4-cjn-10.1177_08445621211058328.pdf]

Please use Adobe Reader or Adobe Acrobat to complete the form

PERMISSION REQUEST (online-fillable form)

Author: *Please complete the first three sections below and forward this request to the Copyright holder.*

1. Parties to the Agreement:

Copyright holder: Dr. Danielle D'Amour, danielle.damour@umontreal.ca, PO Box 6128, Centre-ville Station Montréal, Québec, Canada, H3C 3J7

(Enter name, email address, and address of rights holder above)

Requestor: Suzanne Braithwaite, 12sl40@queensu.ca, 92 Barrie Street Kingston, Ontario K7L 3N6, Canada

(Enter name, email address, and address of author above)

2. Materials: Please enter full citation information for the requested materials, leaving any fields that are not applicable blank.

Figure/Table number(s): Table 1

Publication (Article/Chapter) title: Measuring Actual Scope of Nursing Practice: A New Tool for Nurse Leaders

Author(s): D'Amour, Danielle; Dubois, Carl-Ardy; Déry, Johanne; Clarke, Sean; Tchouaket, Éric; Blais, Régis; Rivard, Michèle

Journal/Book title: The Journal of Nursing Administration Volume/Issue: 42(5)

Article DOI: 10.1097/NNA.0B013E31824337F4 Publication Year: 2012

3. For inclusion within, or in relation to, the article tentatively titled:

Measuring Scope of Practice Enactment among Primary Care Registered Nurses (the "Article")

to be published in: Canadian Journal of Nursing Research (the "Journal")

Journal's estimated individual & institutional print subscribers: < 1,500

Journal's Language of Publication: English and additional languages listed here, if any: ________________

Open Access Disclaimer: ☐ If this box is checked, the Article will be published on an open access basis and may be freely accessed, and may in some cases also be copied, republished, and adapted by others. The Materials will be published with a credit line indicating your ownership of the rights so that readers will have notice that any use of the Materials separate from the Article will require your permission.

4. Nonexclusive Grant of Rights:

By signing this request, you confirm your permission to include the Materials within or in relation to the Article. The permission granted hereunder includes the nonexclusive right throughout the world, in all formats, media, platforms, and channels now known or later developed, to: reproduce, reprint, distribute, transmit, and display the Materials: (i) within the Article, including within excerpts of the Article and within revisions and other derivative works based on the Article published by the Journal owner and its assignees and licensees, (ii) as part of supplemental material related to the Article or derivatives thereof, and (iii) as part of the marketing and promotion of the Article or Journal. The nonexclusive rights granted hereunder in no way restrict your further publication or license of the Materials in any form.

5. Copyright holder to complete:

If you request a specific credit line, please enter this here: ________________

____

Please sign below to confirm your agreement to the terms described above.

Signed Signature numérique de member: 8E3D7BDE-A66A-4E91-9B79-B3856533DFC1 DF2A3040-90CB-466A-9519-383368F77348 Date : 2021.10.11 11:12:56 -04'00' Date 11 OctObre 2021

Name Danielle D'Amour Title Professeure émérite, Université de Montréal
